# Relationship between the Frequency Magnitude Distribution and the Visibility Graph in the Synthetic Seismicity Generated by a Simple Stick-Slip System with Asperities

**DOI:** 10.1371/journal.pone.0106233

**Published:** 2014-08-27

**Authors:** Luciano Telesca, Michele Lovallo, Alejandro Ramirez-Rojas, Leticia Flores-Marquez

**Affiliations:** 1 Institute of Methodologies for Environmental Analysis, National Research Council, Tito, Italy; 2 ARPAB, Potenza, Italy; 3 Departamento de Ciencias Básicas, Universidad Autónoma Metropolitana, Azcapotzalco, México; 4 Instituto de Geofisica, Universidad Nacional Autónoma de México, México D.F., México; Wake Forest School of Medicine, United States of America

## Abstract

By using the method of the visibility graph (VG) the synthetic seismicity generated by a simple stick–slip system with asperities is analysed. The stick–slip system mimics the interaction between tectonic plates, whose asperities are given by sandpapers of different granularity degrees. The VG properties of the seismic sequences have been put in relationship with the typical seismological parameter, the *b*-value of the Gutenberg-Richter law. Between the *b*-value of the synthetic seismicity and the slope of the least square line fitting the *k*-*M* plot (relationship between the magnitude *M* of each synthetic event and its connectivity degree *k*) a close linear relationship is found, also verified by real seismicity.

## Introduction

The transformation from time series to networks allows investigating the time dynamics of complex systems focusing on their topological properties. Zhang and Small [1] constructed complex networks from pseudoperiodic time series by representing each cycle as a basic node. Xu et al. [2] focused on the local properties of complex networks and investigated the distribution of subgraphs within the networks. Gao et al. [3] proposed a phase-space complex network to investigate chaotic time series and then developed a novel multivariate recurrence network [3], [4] for analyzing multivariate time series. Gao et al. [3], [4] deeply studied the features of “network of networks” and successfully uncovered the complicated flow behavior of multiphase flow.

The investigation of time series mapped on networks or graphs by using the visibility graph (VG) method was presented by Lacasa et al. [5]. By means of such mapping, the dynamical properties of time series are converted in topological properties of networks; vice versa, information about time series can also be deduced analysing the characteristics of networks.

In the VG approach a segment connects any two values of the series that can be seen by each other, meaning that such segment is not broken by any other intermediate value of the series. In terms of graph theory, each value of the time series represents a node, and two nodes are connected if there is visibility between them. The mathematical definition of the visibility criterion ([5]) can be given as follows: two arbitrary data values (*t_a_, y_a_*) and (*t_b_, y_b_*) are visible to each other if any other data (*t_c_, y_c_*) placed between them fulfils the following constrain:
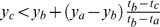
(1)


Let's indicate with *k_i_* the connectivity degree, which is the number of connections of each node *i*. The following properties hold: 1. Connection: each node is visible at least by its nearest neighbours (left and right); 2. Invariance under affine transformations (rescaling of both axes and horizontal and vertical translations) of the time series. 3. Directionality: the link could have or not direction; in case of absence of directions any value could be visibly linked with previous and following values; in case of directionality each value could be visibly linked with only the previous or following values [6].

It was shown that the graph developed using the VG method transforms periodic, random and fractal time series into regular, random and scale-free networks respectively [5], [7], [8].

**Figure 1:**
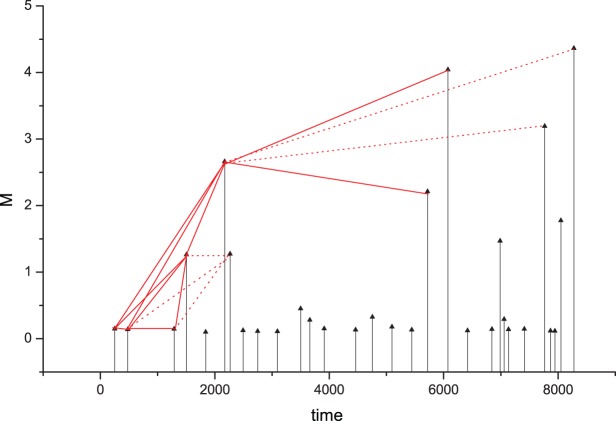
Sketch of the VG method. The black vertical arrows indicate the events; the height of each arrow is proportional to the magnitude. The magnitudes represent the nodes of the graph. The solid visibility rays (red lines) between the events define the permitted links connecting the nodes; the dotted ones define the not permitted links.

Recently an application of the VG method to the analysis of earthquake magnitude time series was performed by Telesca and Lovallo [9] in relationship with the Italian seismic catalogue. The time series {*y_i_*} was represented by the point process of the earthquake magnitudes, and the VG method was applied as shown in Fig. 1. Their findings pointed out to the presence of power-law behaviour in the distribution of the degree *k*. They also found that the form of the degree distribution is independent of the time-clustering structure, and of the increase of the magnitude threshold.

Telesca et al. [10] applied the VG method to the sequences of earthquakes occurred in the sub-duction zone of Mexico and found that the *k*-*M* plots (which is relationship between the magnitude *M* of each event and its connectivity degree *k*) were characterized by increasing trend of *k* with the increase of *M*, revealing, thus, the property of hub as typical of the higher magnitude events. Computing the slope of the line fitting in a least square manner the *k-M* plot, an experimental relationship was hypothesized between the slope of the *k-M* relationship and the *b*-value of the Gutenberg-Richter law: lower the *b*-value, lower the slope of the *k-M* plot. It was, then, deduced that the VG properties of seismicity can resemble the seismological properties described by the Gutenberg–Richter law. Additionally, the VG method, since it does not take into account only the magnitudes of the events, but also their time occurrence, could suggest a way to analyze the statistical properties of seismicity more general than the Gutenberg–Richter law.

In the present paper, we investigate the relationship between the *b*-value of the Gutenberg-Richter law and the slope of the *k-M* plot as obtained by the VG method on the synthetic seismicity generated by a simple stick–slip system with asperities.

## Experiments

**Figure 2:**
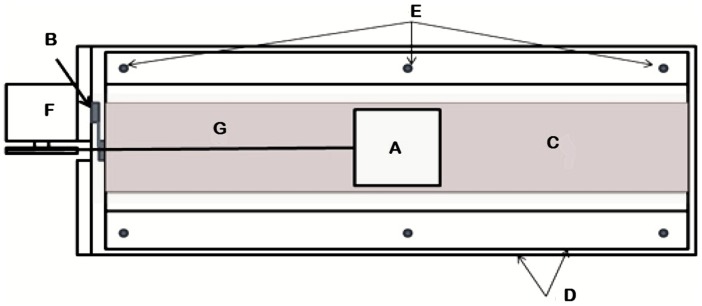
Experimental stick-slip block system with asperities [22]. See text for details.

Our experiments were aimed to simulate the interaction between two fault planes with asperities. Thus, we build up a frictional system, namely in the stick–slip process of spring–slider setup, subjected to a mechanical forcing. The spring–slider system is considered as a proxy of geological faults under tectonic stress. The experimental setup, designed by Vargas et al. [11], [12], which is characterized by two sandpapers in relative movement mimicking the relative motion of two tectonic plates. This experimental device, based on the classical model proposed by Burridge and Knopoff [13], is schematically shown in Fig. 2. The system consists of an aluminum block (A) of 0.1 m length, 0.1 m width, 0.025 m height, and 0.5 kg mass, which slides over a frictional surface with asperities (C), consisting of an aluminum track of dimensions 0.7 m length, 0.22 m width and 0.003 m height. The inferior surface of the block (A) and the aluminum track (C) are coated with sandpapers with different granularity degrees. Below the aluminum track, a low friction suspension system consisting of two glass plates was settled. The superior glass plate has a thickness of 0.009 m and rests on a set of steel spheres (E), with diameter of 0.004 m, which can roll over a second glass plate of 0.012 m thickness (D). All the suspension system is placed over a metallic frame to maintain it in a leveled position. The object (B) is a charge cell (Omega LCL), which works as a bumper against the metallic frame and allowing recording the force exerted by the inferior plate over the cell when the elastic rope (G) is kept in tension. The rope is a fishing string with a diameter of 5×10^−4^ m and a charge limit of 8.0 kg. The rope connects the aluminum block with the motor (F) through a pulley. To pull the block a CD-motor is used with speed control (Baldor CD5319) with two gearboxes of 60/1 in series connection. During the experimental runs the string is pulled with a constant speed of 0.0133 m/min resulting in a block displacement of about 0.6 m in 45 min. The charge cell was polarized by means of a regularized power source of 10 DCV; the cell sensitivity is 2 mV for each volt from the power source. In the experiment the maximum charge is 20 N, producing a signal of 20 mV. The signal of the charge cell was registered with a resolution of ±0.5×10^−6^ V by means of a digital voltmeter (Keithley 197A), and connected to a personal computer through a GPIB interface using a QBasic program. It was used the voltmeter minimum sampling time of 0.33 s. With regard the interaction between the two surfaces, the different asperities are calibrated by means the sandpaper grade, low grade means high asperity. For each experiment, the data set were obtained by using combinations of different sandpaper granularity degrees, classified in agreement to the European Financial Planning Association (EFPA) (www.efpa-europe.org/) standard abrasives (sandblasting material abrasive 16 mesh Fandelli International Corporation commercial trademark, Houston Texas). In our experiments, we coated the inferior surface of the moving block with sandapaper of granularity degree 36, while the aluminum track was coated with sandpapers of different granularity degrees: 40, 50, 60, 80, 100, 180 and 220. Both rugged surfaces were in opposition to each other. For each combination we collected two datasets of the synthetic seismicity: after the first run (R1) and after the fifth run (R5), mimicking respectively the absence or not of sediments between tectonic plates [14], [15].

## Results and Discussion

**Figure 3:**
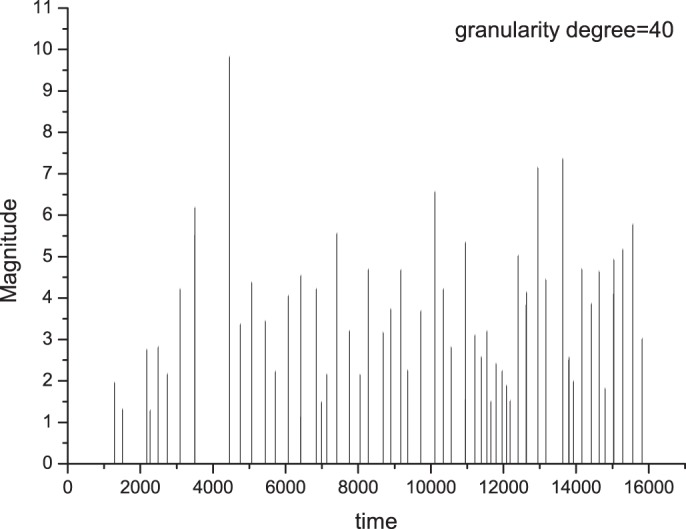
Example of synthetic seismicity obtained during one run.

**Figure 4:**
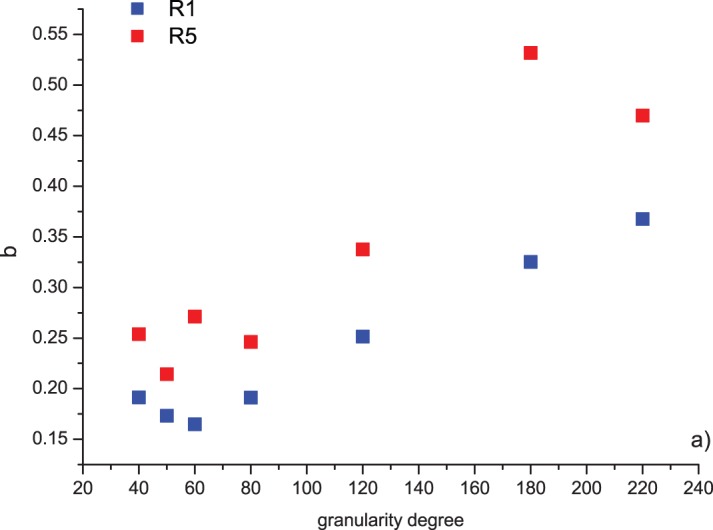
Relationship between the *b*-value, the run and the granularity degree of the aluminum track (the sandpaper coating the inferior surface of the moving block has granularity degree 36 in all the experiments). The first run is indicated by R1, while the fifth one by R5.

We analysed the synthetic seismicity generated by the experimental stick–slip system as described above. During a run, a displacement of the block simulates an earthquake and its length corresponds to the magnitude of the earthquake. Therefore, firstly, we normalized the displacements in any experimental run to be within the arbitrary range of 1–10, thus, simulating the seismic magnitude of the events. The length of datasets varies from 116 to 459 events. Fig. 3 shows an example of synthetic seismicity. We considered the events with threshold magnitude of 1. For each synthetic seismic sequence, the *b*-value of the Gutenberg-Richter law was calculated by using the maximum likelihood estimation method [16]:
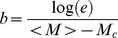
(2)where <*M*> is the average magnitude and *M_C_* is the completeness magnitude of the seismic sequence representing the minimum magnitude over which the frequency-magnitude distribution behaves as a power-law, *N*∼10^-*bM*^. In our case *M_c_* corresponds with the minimum magnitude *M_min_* of the synthetic sequence. Fig. 4 shows the relationship between the *b*-value, the run and the granularity degree. It can be observed that the *b*-value obtained for the first run R1 is lower than that obtained for the fifth run R5 for each granularity degree. The *b*-value controls the proportion of earthquakes with different magnitudes [17], therefore a higher *b*-values indicates a relatively higher number of small events than large events and vice versa. In our experiments, the magnitude of the events is the length of the displacement during a slip. This indicates that during R1 the proportion of longer displacements respect to the smaller ones is higher than during R5. If R1 and R5 would indicate respectively a younger and a mature fault, this result suggests that larger events are more probably produced by a younger fault than a mature one. As shown by the experiments by Renard et al. [18] on a halite (NaCl) slider held under constant normal load and dragged across a coarse sandpaper substrate, the cumulative slip produces a gradual decrease in the friction coefficient also due to the accumulation of gouge particles that dissipate strain by a rolling effect. Furthermore, it can be observed that for low granularity degrees (until 80), *b* fluctuates around a mean value regardless of the run, but for higher granularity degrees the *b*-value increases with the granularity degree. Higher granularity degree means lower roughness; and, lower roughness corresponds to a higher *b*-value, which indicates a relatively higher number of small events than large ones. This result is consistent with the previous one of the increase of the *b*-value with the run. For the same run, the rougher surfaces (low granularity degree) are able to produce larger displacements during the slip. The mechanism responsible of such phenomenon could be due to the presence of asperities that can pin the interface during sliding, producing an augmenting of the local shear stress so that when this becomes larger than the friction the block slides with a length proportional to the stress drop, similarly to what happens for real faults [19]. If the granularity degree is lower, and so the roughness higher, the sliding wedges the asperities on the inferior surface of the block into the asperities of the track, and this would correspond to a higher stress along the slip direction; while if the granularity degree is higher, and so the roughness lower, the interface “wedging” phenomenon of the asperities during sliding is weaker, and also weaker is the stress along the slip.

**Figure 5:**
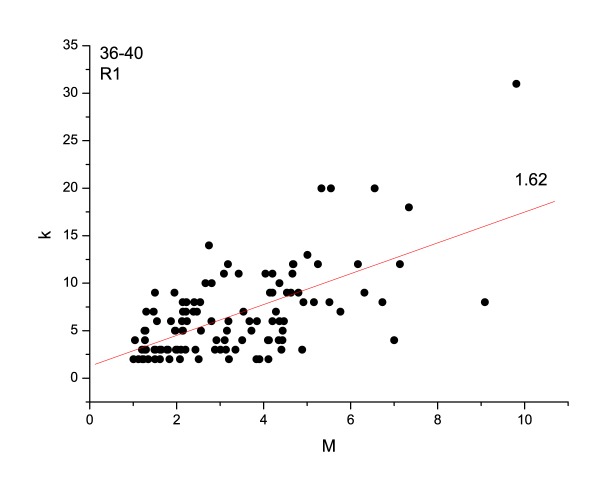
*k-M* plot and LSM fitting line during run R1.

**Figure 6:**
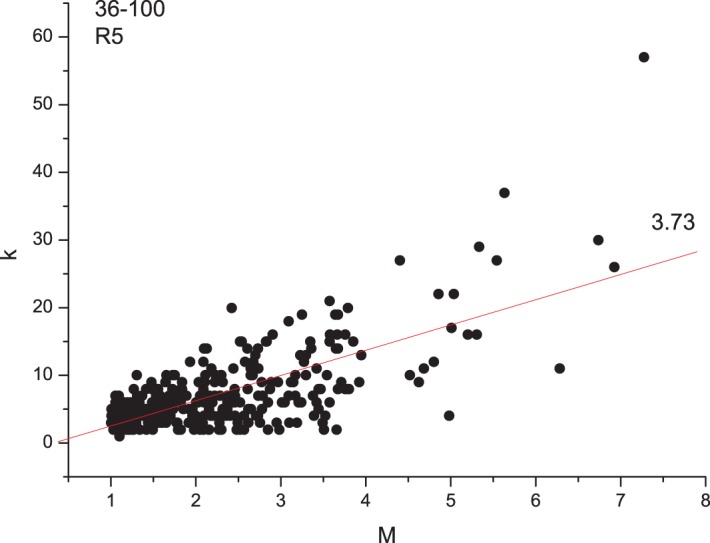
*k-M* plot and LSM fitting line during run R5.

**Figure 7:**
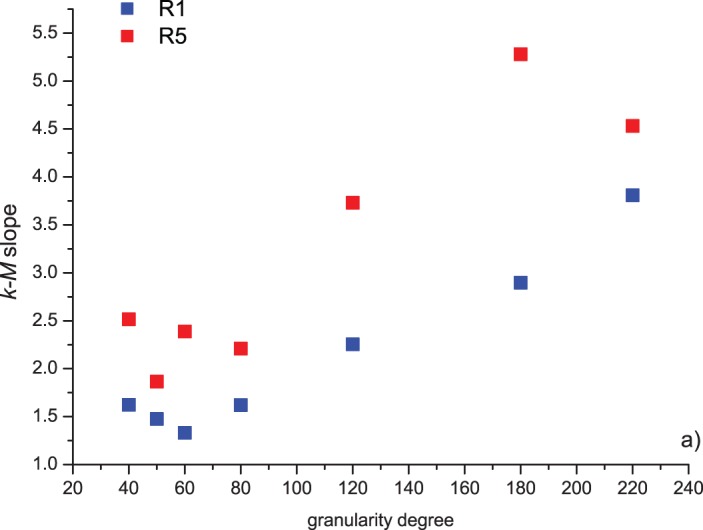
Relationship between the *k-M* slope (of the LSM fitting line), the run and the granularity degree of the aluminum track (the sandpaper coating the inferior surface of the moving block has roughness degree 36 in all the experiments). The first run is indicated by R1, while the last fifth one by R5.

Figs. 5 and 6 show the relationship between the connectivity degree *k* and the magnitude *M* of the event (*k-M* plot) for two representative cases. The line fitting the cloud of points by the least square method (LSM) is also shown. Fig. 7 shows the relationship between *k-M* slope (of the LSM line fitting the plot), the run and the granularity degree. The behavior is similar to that observed for the *b*-value: for low granularity degrees (until 80), the *k-M* slope has a fluctuating behavior; for higher granularity degrees, and so lower roughness, it increases with the increase of the granularity degree for both runs. Also the *k-M* slope is higher for R5 than those for R1.

The lower value of the k-M slope for rougher surfaces indicates that events with the same magnitude have a lower connectivity if produces by the sliding of a block coated by a rougher sandpaper with lower granularity degree. This phenomenon can be reasonably explained by the role played by the larger events, mostly produced during the sliding of rougher surfaces. In fact, larger events not only play the role of “hub”, being “visible” by all the neighbors, but also prevent the smaller events (located before and after the “hub”) to connect with each other; therefore the events produced during the sliding of rougher surfaces would have higher probability to have larger connectivity if the higher ones would not impede their “visibility”.

**Figure 8:**
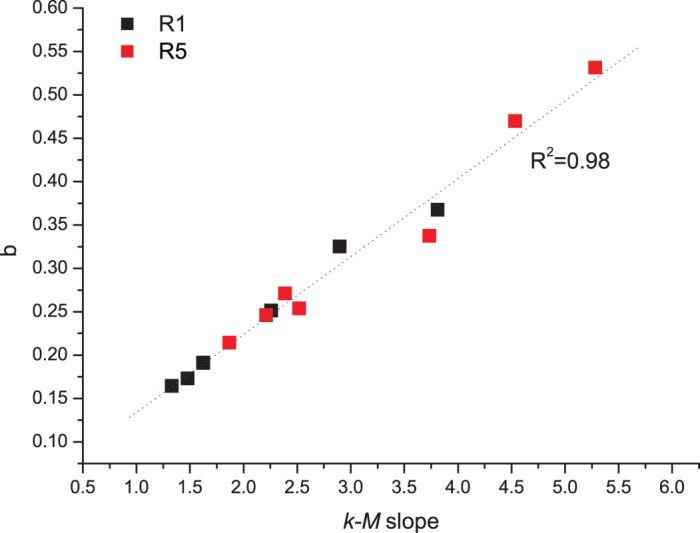
Relationship between the *k-M* slope and the *b*-value for the synthetic seismicity during the first (black squares) and the fifth run (red squares). The linear correlation is very good (*R* ^2^∼0.98).

**Figure 9:**
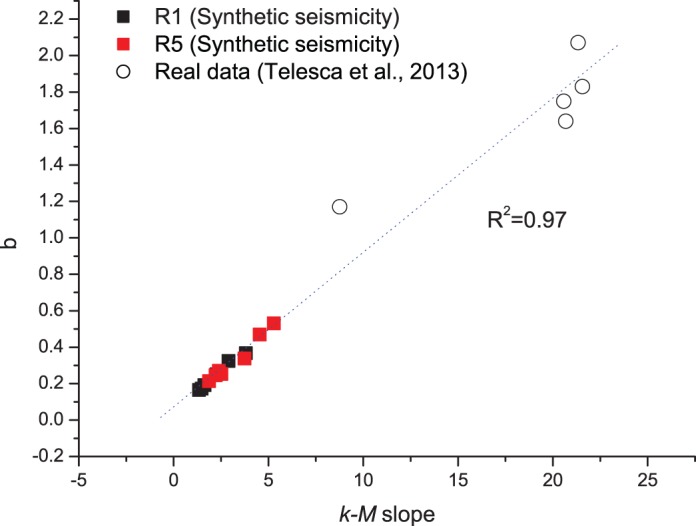
Relationship between the *k-M* slope and the *b*-value for the synthetic seismicity (black and red squares) compared with that for the real seismicity data (white circles) obtained by Telesca et al. [10]. The linear correlation is very good (*R* ^2^∼0.97).

Fig. 8 shows the relationship between the *b*-value and the *k-M* slope; the linear correlation between the two parameters is very good (*R* ^2^∼0.98). In Fig. 9 the relationship shown in Fig. 8 concerning the results of the synthetic seismicity, is enriched by the results obtained by Telesca et al. [10] on real seismicity data recorded in five seismic areas of the Mexican sub-duction zone: the linear correlation between the *b*-value and the *k-M* slope is very good as well (*R* ^2^∼0.97). The correlation found between the topological properties (*k-M* slope) and the seismological properties (*b*-value) of earthquake sequences (synthetic and real) confirms the utility of the visibility graph in analyzing observational time series. More specifically, it is reinforced the idea of earthquakes as constituting a network, in which few events (with the higher magnitudes) play the role of “hub” with many links, while many others (with the lower magnitudes) have less links.

## Conclusions

The study presented in this paper shows the relationship between the slope of the LSM line fitting the VG *k-M* plot of the seismic network and the seismological Gutenberg-Richter *b*-value. Both the synthetic seismicity obtained by a laboratory experiment (with different granularity degrees and different runs) and the real one recorded in five seismic areas of Mexico verify such relationship.

The VG analysis of the seismic networks could be considered an alternative way of investigating the earthquake magnitude sequences, different from the standard one based on the frequency-magnitude distribution along with its best fitting with a proper distribution law (recently, non-extensive statistical distribution laws have been proposed to fit the frequency-magnitude distribution alternatively to the well-know Gutenberg-Richter law ([20], [21]). Furthermore, the VG method could represent a more general way to analyze the earthquake magnitude distribution, because it takes into account not only the magnitude (as the standard frequency-magnitude distribution analysis and its classical Gutenberg-Richter law do) but also the time occurrence of the events, due to the connectivity law (Eq. 1) by which the seismic events are linked with each other; this suggesting that the classical Gutenberg-Richter law could be considered as a particular case.
